# Convolutional Neural Networks for Challenges in Automated Nuclide Identification

**DOI:** 10.3390/s21155238

**Published:** 2021-08-03

**Authors:** Anthony N. Turner, Carl Wheldon, Tzany Kokalova Wheldon, Mark R. Gilbert, Lee W. Packer, Jonathan Burns, Martin Freer

**Affiliations:** 1 School of Physics and Astronomy, University of Birmingham, Edgbaston, Birmingham B15 2TT, UK; c.wheldon@bham.ac.uk (C.W.); M.Freer@bham.ac.uk (M.F.); 2 UKAEA, Culham Science Centre, Abingdon, Oxfordshire OX14 3DB, UK; m.gilbert@ukaea.uk (M.R.G.); lee.packer@ukaea.uk (L.W.P.); 3 AWE PLC, Aldermaston, Reading RG7 4PR, UK; Jon.Burns@awe.co.uk

**Keywords:** radio-isotope identification, convolutional neural network, gamma spectrometry, simulations, GEANT, nuclear applications

## Abstract

Improvements in Radio-Isotope IDentification (RIID) algorithms have seen a resurgence in interest with the increased accessibility of machine learning models. Convolutional Neural Network (CNN)-based models have been developed to identify arbitrary mixtures of unstable nuclides from gamma spectra. In service of this, methods for the simulation and pre-processing of training data were also developed. The implementation of 1D multi-class, multi-label CNNs demonstrated good generalisation to real spectra with poor statistics and significant gain shifts. It is also shown that even basic CNN architectures prove reliable for RIID under the challenging conditions of heavy shielding and close source geometries, and may be extended to generalised solutions for pragmatic RIID.

## 1. Introduction

Radio-Isotope IDentification (RIID) finds a broad scope of applications in security, decommissioning, and public health and safety. Each come with their own challenges, but the effectiveness of any detection system stems from its physical limitations and algorithm performance. The ubiquity of HPGe and NaI detectors across the nuclear industry establishes a consistent landscape that is ideal for RIID development, keeping physical constraints relatively predictable.

In contrast to advances in detector materials, identification algorithms are constantly developed and updated. Persistent challenges in the explicit handling of transient effects (such as gain drift) have led to new approaches with machine learning [1,2,3,4,5,6]. By definition, these models operate without being explicitly programmed, instead making decisions based on generalised rules born of experience.

Artificial Neural Networks (ANN) have dominated the deep learning RIID approaches since their inception [1]. These typically ‘fully-connected’ models have a huge number of trainable parameters for even simple networks. Efforts must consistently focus on reducing the number of input features given to the model, often discarding information. Several interesting approaches for extracting underlying signals from background noise include Principle Component Analysis [2,7] and the closely related K-L transform [5]. Others rephrase the problem, performing feature extraction manually [1,3,4,6] and providing these as input data instead of raw spectra.

A variant known as the Convolutional Neural Network (CNN) may provide new directions [8,9,10] that inherently mitigate many of the issues ANNs suffer from, while possessing unique advantages beneficial to the RIID problem space. This work explores the application of sequential CNN models as pragmatic RIID solutions, able to address the challenges of transient effects and shielded sources.

## 2. Spectra for Training and Testing

Any machine learning technique requires training, validation, and test data to work with. A generalised gamma spectrometry simulator was developed for high-fidelity modelling of a diverse range of data sets. The details of this GEANT4-based [11] simulator are available in Ref. [12]. The synthetic spectra generated are used to train CNN models, monitoring for undesirable effects, such as over-fitting. Section 5.4.1 later investigates the addition of real experimental spectra to training sets.

A 3 MeV range was simulated for nine sources that are readily available to this work: ${}^{108m}$Ag, ${}^{241}$Am, ${}^{133}$Ba, ${}^{207}$Bi, ${}^{60}$Co, ${}^{137}$Cs, ${}^{152}$Eu, ${}^{22}$Na, and ${}^{44}$Ti. To demonstrate that these models may be used, even where experimental spectra are absent, the reference model (Section 5.2) was trained entirely on simulated data, then tested on unseen experimental data.

Unfortunately, even when simulated, any comprehensive training set would take months to produce if all variations in physical effects, geometries, source combinations, and shielding scenarios were to be included. By simulating ‘template’ spectra instead, physically accurate variations in gain shift, count rate, and relative activity of multiple isotopes can be randomly varied to make new, equally valid training spectra. The analogy to image data is changing colour balances or rotating the original: a common practice in data science.

### 2.1. Template Spectra

Normalised template spectra were combined into multi-isotope spectra with an experimental background for every unique combination of sources. Each template contributes a share of the total counts based on randomised factors (0.2–1.0). To provide significantly more training spectra with poorer statistics in the ∼10${}^{3}$–10${}^{6}$ counts range, this total was sampled from a logarithmic scale. A reasonable minimum of 500 counts per source was ensured. Background radiation was capped at 25% of the total counts from all other sources.

**Figure 1:**
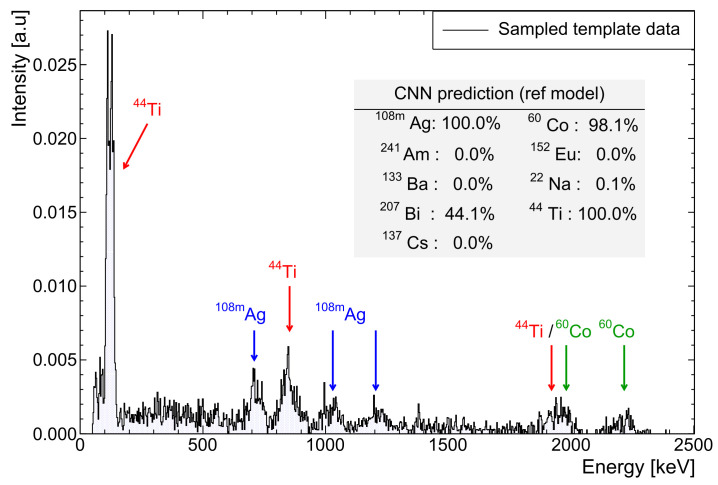
Example 4 × 10${}^{3}$ count NaI spectrum (7.05(1)% @662 keV) containing natural background, ${}^{108m}$Ag, ${}^{44}$Ti and ${}^{60}$Co. A 31 keV threshold and gain shift of ×1.7 are applied. Note the ambiguity in model predictions (see Section 5.2) for ${}^{207}$Bi, which is extremely similar in profile to ${}^{44}$Ti.

Lower energy thresholds were applied with a 40 keV maximum for the sake of ${}^{241}$Am, which predominantly emits a 59.5 keV γ-ray. A small lower threshold is often used in experimental measurements to exclude low level noise, but 40 keV is large enough to remove significant features from several spectra. Gain shifts were implemented for a large 0.5–2.0 range of factors, emulating extreme transients in temperature or signal processing electronics to test robustness. The resulting spectra were re-binned, using interpolated counts to ensure a consistent channel structure, with photopeaks now shifted from the calibrated energy. Figure 1 shows an example of these randomised perturbations.

Finally, the spectra were compressed from 8192 to 1024 channels to clarify the features in those with poor statistics, normalising the intensities to unity for consistency. The same process was applied to templates of experimentally obtained data, though the background must be removed before sampling. All data pre-processing used the ROOT [13] framework. For training data load into memory, a data generator was used for scalability. With this, a buffer of several batches is loaded in parallel while the model is training.

### 2.2. Test Spectra

**Figure 2:**
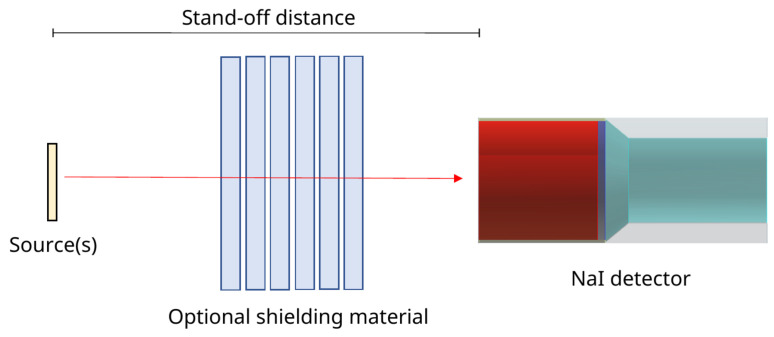
Diagram of the basic test scenario. A 3${}^{\prime \prime }$× 3${}^{\prime \prime }$ NaI detector was placed 1, 5, and 10 cm from the source, biased to +800 V. The signal was passed through an Ortec 571 amplifier (2 μs shaping time) and digitised with an Ortec Easy-MCA-8K Multi-Channel Analyser (MCA). Metal plates were placed between the source and detector for shielding.

For testing, data sets were prepared for a 3${}^{\prime \prime }$× 3${}^{\prime \prime }$ NaI detector at 1, 5, and 10 cm from the source to investigate the close geometry effects. For shielded scenarios, up to 6 cm of aluminium shielding was placed between the source and detector, with the source fully obscured (see Figure 2). All simulated scenarios have four training sets and six test sets of experimental data for performance testing, each using unique random seeds for including further perturbations. The quoted classification rates and results are taken as the average across all test sets.

## 3. Convolutional Neural Networks

Originally designed for image classification, the core of a CNN optimises successive ‘filter’ sets that are convolved with an input image. ANNs consider every pixel to be independent, with each pixel connected to every part of a hidden layer. Complexity, therefore, rapidly increases with scale. By comparison, CNN convolution layers train only a set of small (typically 3 × 3) filters instead. The computational expense is significantly reduced, scaling almost linearly with the number of input samples. Context is also considered by including information from neighbouring pixels. Applying this architecture to gamma spectra, where many important ‘features’ (such as photopeaks) are localised over channels, is very beneficial.

By convolving a gamma spectrum with filters, new abstractions of features are created. The output tensor of each convolution becomes a ‘feature map’. More complex features are learnt by building upon multiple convolution layers. The first layers typically end up performing basic operations, such as edge detection.

**Figure 3:**
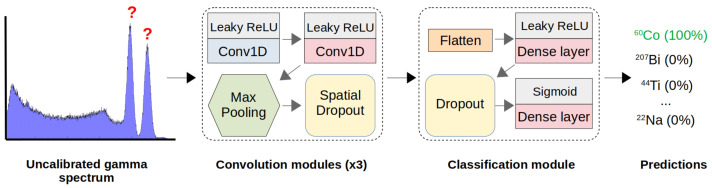
Simplified CNN structure for radio-isotope identification. The sequential model consists of several convolution modules, each performing 1D convolution operations to extract relevant features. For classifications, the resulting tensor is flattened into a vector to interface with a small, fully-connected ANN. The final output layer provides the probability of each source being present in a gamma spectrum.

Since filters are optimised during training, the model learns to extract features and decides which are important for the final identification. This places the responsibility of feature extraction on the model with minimal manual pre-processing. A small, fully-connected structure following the convolution operations provides the probability of each source being present in the spectrum. An overview of the model architecture is shown in Figure 3. Presented in this work is an approach to RIID, using ‘multi-class, multi-label’ classification based on the special 1D case of a CNN. An overview of this network type is provided in the next section, with key choices concerning certain parameters discussed in the results (Section 5).

## 4. Methods

### 4.1. Model Definition and Learning

The rapid uptake of machine learning methods in the last five years has been driven by accessibility. This work utilises the open-source TensorFlow [14] libraries. Such powerful tools would otherwise require a great deal of effort to implement—another limiting factor in the complexity of early models, following Olmos et al. [1].

**Figure 4:**
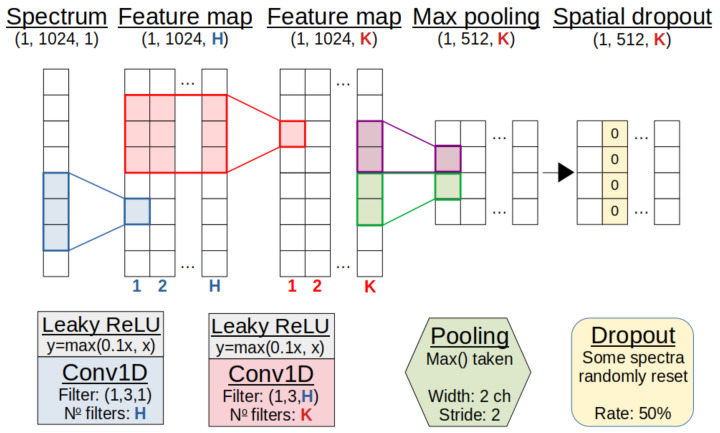
Representation of the convolution module. An array of intensities represents the spectrum. This trains first *H* filters, then *K* filters in two convolution layers to emphasise key features in the spectra. Pooling and dropout then reduce the dimensionality and combat over-fitting. For demonstration purposes, the shapes exclude a dimension for batch size. See text for details.

This CNN model consists of groups of 1D convolution layers in three modules (Figure 4), each with many filters. By stacking two convolution operations in each module, a larger channel range is seen with fewer parameters, while intermediate activations exaggerate weights to make features more expressive. These activation functions introduce non-linearity into the model, with Rectified Linear Unit (ReLU) based functions (y=max(0,x)) chosen over legacy functions, due to their robust nature.

The ‘max pooling’ operation down-samples convolved spectra by propagating only the maxima of non-overlapping channel groups. This reduces the size of the output tensors while retaining important features. ‘Spacial dropout’ is also employed, removing spectra from feature maps at random to prevent over-dependence on any particular path for a final classification.

**Figure 5:**
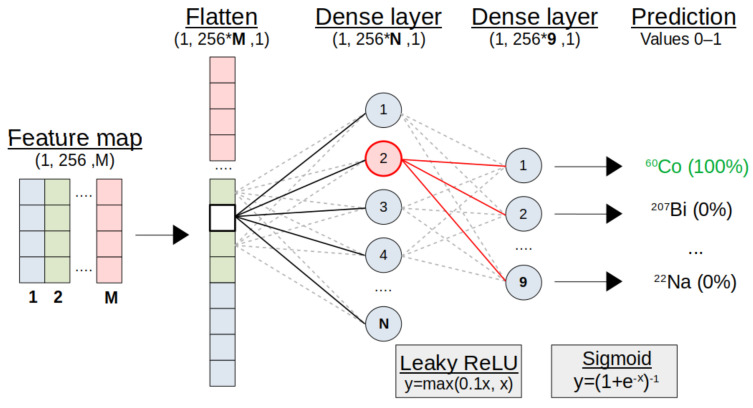
Representation of the classification module. The convolution module output is flattened into a vector. A small ‘fully-connected’ ANN makes final predictions of present sources, with a 0–1 probability through Sigmoid activation. See text for details.

With the tensor of the final convolution module flattened into a vector, classifications are decided through a small ANN (Figure 5). This may be only two fully-connected or ‘dense’ layers, again randomly resetting individual nodes with dropout to discourage over-fitting. The final layer and its activation function decide the type of classification.

For practical RIID with a single model (Figure 3), this is a multi-class, multi-label problem. Spectra contain arbitrary combinations of multiple labels, each corresponding to one of the nine unique classes (sources). Arrays of labels are encoded to designate sources as either present (1) or not present (0) for each spectrum. The model is compiled with the Adam optimiser [15] and a binary cross-entropy loss function [16]. All parameters relating to the model structure and compilation are known as ‘hyperparameters’, with specific examples listed in the results of Table 2.

### 4.2. Performance Evaluation

Evaluation of this multi-label classification problem is best described by building upon binary concepts. The final Sigmoid activation ($y={\left(1+e^{-x}\right)}^{-1}$) encodes the probability of each source being present in a test spectrum. Models are trained on a decision threshold of 0.5, with probabilities >0.5 defining a ‘positive’ (1) classification. If the source is actually present, it is correctly identified as a ‘True Positive (TP)’. Correspondingly, the other possible outcomes are shown in Table 1.

Performance may vary as this decision threshold changes. For the balanced training sets used, a Receiver Operator Characteristic (ROC) provides a good indication of performance with threshold. To produce a ROC plot, True Positive Rate (TPR) and False Positive Rate (FPR) pairs are plotted for each threshold (t), defined as follows:

(1) $$TPR=\frac{TP}{TP+FN},\;\mathrm{and}\;FPR=\frac{FP}{FP+TN}.$$

For RIID applications, the TPR is a measure of ability to find all sources present, while FPR is the probability of a false alarm. A closely related metric is ‘precision’, or how relevant the predictions are.

(2) $$precision=\frac{TP}{TP+FP}$$

The ROC curve is extended to the multi-label case by considering each label individually and together as an average. This information may be summarised in a single value by the Area Under Curve (AUC) as follows:

(3) $$AUC=\int_{-\infty }^{\infty }TPR\left(t\right)FPR^{\prime }\left(t\right)\;dt.$$

The ‘Perfect’ Prediction Rate (PPR) is also used in this work as a complementary measure of the RIID performance, given the real world applications. For example, averaging rates over the many sources may fail to reflect a consistently poor performance when making predictions for one in particular. Predictions are only considered perfect when every possible source is correctly identified as either TP or TN, acting as a much harsher performance metric. The PPR is simply the fraction of the test spectra that had such perfect overall predictions.

## 5. Results and Discussion

The performance of any RIID model based on machine learning is dependent on two primary aspects. First, the model’s architecture and hyperparameters must be chosen with desirable properties in mind. One example is the dropout rate of a dense layer, where a balance must be struck between dropping so few nodes that over-fitting is a risk, or so many that performance suffers. The second consideration must be the data sets used to train the model. For example, larger data sets are generally better for deep learning because the model has more representative samples to learn from. An investigation of both aspects was carried out. The same model architecture dubbed the ‘reference model’ was used to evaluate all data sets, including the challenges of heavy shielding and close geometries.

### 5.1. Reference Model

For every unique combination of the nine sources, only 20 spectra were produced to build a small, entirely simulated training set for the 3${}^{\prime \prime }$ NaI detector at 10 cm. Relatively few spectra (∼1 × 10${}^{4}$) means that if the model does not generalise well, performance will suffer. Significant over-fitting will also make poor predictions on the unseen real data. Several equally small test sets were produced from the experimental spectra, provided to the models as uncalibrated spectra with no pre-processing beyond rebinning to 1024 channels and count normalisation.

Combinations of the two most common optimisers, Adam [15] and RMSProp [17], were assessed with a variety of initialisers. While consistent across initialisers, a difference between the two optimisers saw PPR change by 3.2(1)% on average. The marginally better He normal [18] initialiser was chosen to randomly set initial weights from a truncated normal.

**Figure 6:**
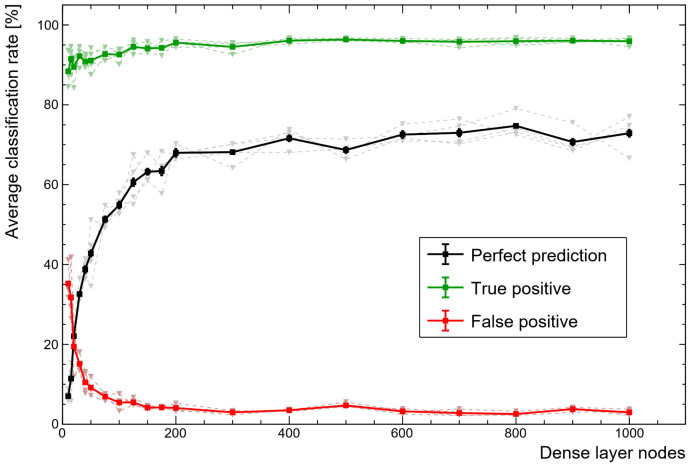
Variation in performance rates as the dense layer size of the classification module is varied. Bold lines represent the average across all training and test sets. Diminishing returns quickly encourage a compromise as dense layers rapidly increase in complexity with scale.

While the second dense layer (Figure 5) must have nine nodes (one for each class), the first can vary and have a significant impact in comparison to the other hyperparameters. Figure 6 demonstrates how important the size of this small ANN is for making good use of the features extracted by the convolution layers. There is a clear degradation in performance when using a smaller ANN, but the computational expense rapidly increases with the number of nodes, thereby encouraging a compromise.

The ‘leaky’ ReLU activation function variant was implemented throughout the model to combat an issue where negative weights may result in a gradient of zero, leading to the well-known ‘dying node’ problem. A small gradient in the negative weights was, therefore, included as y=max(αx,x), where α=0.1, rectifying the problem of vanishing gradients.

A collection of all important hyperparameters used in the final reference model is shown in Table 2. Of course, there are many combinations and drastically different CNN structures possible. However, with the exception of dense layer size, most appropriate hyperparameter ranges showed only minor effects on classification performance during the prototyping phase of this work.

### 5.2. Reference Model Performance

Performing evaluation on the test sets of unseen experimental data, the overall classification rates are shown in Table 3. The average perfect prediction rate across test sets was 74.4(9)%. A precision of 99.61(3)% reflects that few of the possible labels were predicted incorrectly in the remaining test spectra, indicating that correct label predictions were made with a high degree of confidence. The average time to load spectra and make predictions using the pre-trained model was <5 ms per spectrum on an i5-5200U CPU, making real-time updated classifications readily available to an end user.

As discussed in Section 4.2, the rates shown in Table 3 take performance as a whole. Individual binary confusion matrices can be extracted for each class, but the clearest visual aid for observing class performance is the ROC curve.

**Figure 7:**
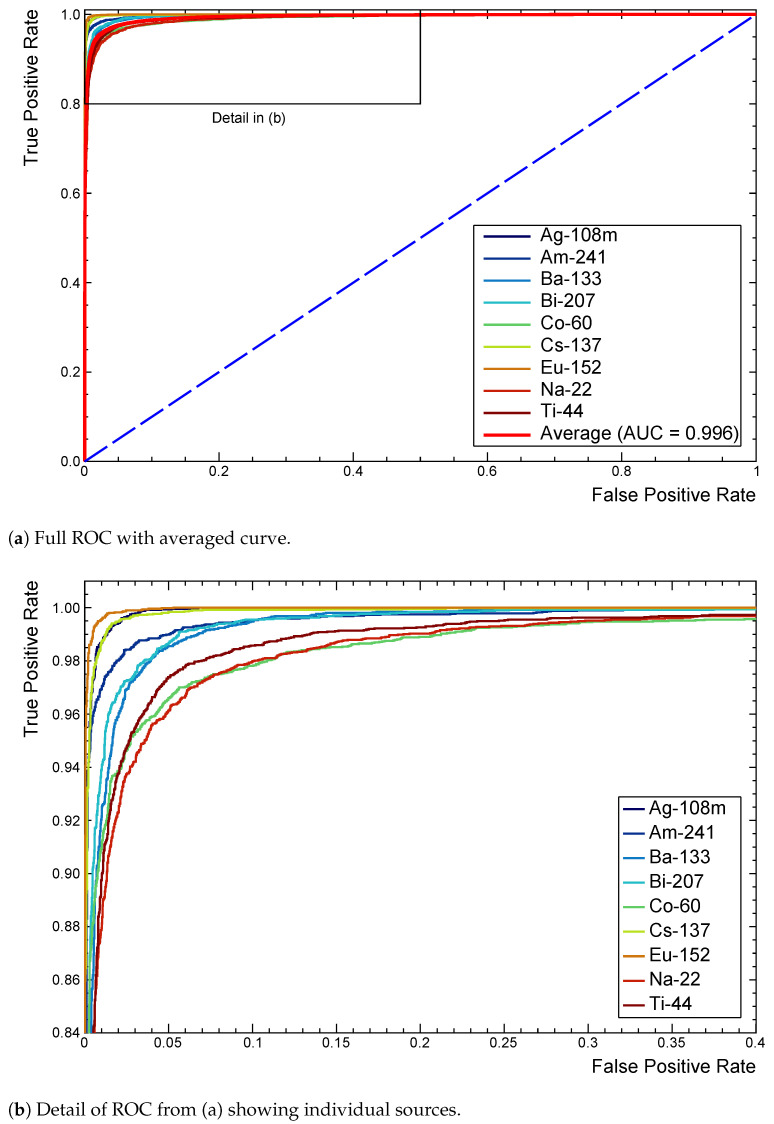
ROC curves extended to the multi-label case for the reference model. Provides comparisons of performance with each source as the decision threshold is varied. The average (red) is included in (**a**) as a reference for model performance across all classes. (**b**) shows ^22^Na, ^60^Co, and ^44^Ti as marginally responsible for more false classifications.

Figure 7a shows a full ROC for this model after extension to the multi-label case. Each curve tends towards (0,1) as performance improves, with the dashed line representing the random assignment of positive or negative classifications.

Inspecting the detail in Figure 7b identifies ${}^{22}$Na, ${}^{60}$Co, and ${}^{44}$Ti as being marginally responsible for more false classifications. Slight differences in AUC confirm this quantitatively. A ${}^{22}$Na spectrum in particular contains very few features, all of which closely overlap with those of many other sources to make distinction difficult. The shallow architecture found ${}^{207}$Bi or ${}^{44}$Ti slightly more challenging, without enough higher level feature extraction, making it difficult to separate the two extremely similar spectra. Figure 1 demonstrates such a result. The ${}^{60}$Co source, while distinctive, has both major peaks at energies that overlap with several other sources. Again, this makes separating the sources in the overall profile much more difficult for even a trained spectroscopist. For RIID, these multi-label ROC curves prove to be an extremely useful tool for quickly recognising which sources different models have difficulty with.

### 5.3. Challenging Conditions

With knowledge of the reference model performance on unshielded sources at a 10 cm stand-off distance, new models were trained from scratch on challenging data sets using the same architecture. Results for the challenging conditions of close geometries and shielded sources are summarised in Table 4.

True coincidence summations become more likely in close geometries. Indeed, the solid angle of the 1 cm distance means that almost all spectra contain additional photopeaks, drastically increasing the complexity of the multi-isotope spectra. Despite this, the reference model was able to perform relatively well, even with the additional complexity. Note that this is in part due to the accurate handling of coincidence summations of the simulator, which produces good training data as a result. The probability for summation reduces quickly with distance, following an inverse square law, so it is unsurprising that the 5 cm and 10 cm stand-off distances performed similarly.

Attenuation of γ rays through shielding is exponential with thickness. Lower energy photons (<100 keV) become increasingly more likely to interact, affecting them more severely. Backscatter, where a photon Compton scatters in material prior to entering the detector, also changes the profile of the spectra significantly. A combination of these effects makes shielded scenarios one of the greatest challenges of RIID.

Heavily shielded conditions were simulated to reproduce experimental test data. Sources were placed 10 cm from the detector, with up to 6 cm of aluminium positioned between the two. The classification rates suffer as shielding is increased. In contrast to other data sets, the ROC curves reveal ${}^{44}$Ti, ${}^{241}$Am and ${}^{152}$Eu to be the cause of more incorrect predictions. Interestingly, it can be inferred that the ${}^{152}$Eu predictions rely quite strongly on the more prominent, low-energy peaks for distinguishing the source.

The ${}^{60}$Co and ${}^{22}$Na spectra, each with prominent features at higher energies, were replaced by those that rely on low-energy features. Of course, these are now attenuated, often to the point of being missing. At 6 cm of Al, there is simply not enough of the solitary ${}^{241}$Am peak left to stand out among the backscatter and other sources, with the average false negative rate of 12.3(3)% being almost entirely due to this source.

While perfect rates drop below 50%, it is important to note that the perfect rate for randomly assigning a label as present is just 0.2% for this set. It is very encouraging that the model performance reflects the challenges of the physical phenomena involved, enabling a more intuitive understanding of model limitations and how pre-processing may be adapted to highlight certain features.

### 5.4. Optimising for Deployment

The CNN approach has demonstrated excellent performance for the challenges addressed, but the focus consistently lay with changes in the model architecture. Pragmatic solutions for real-world deployment cannot ignore the data sets themselves. The key theme is always one of representation. The application of common tactics for improving performance beyond hyperparameters was investigated.

#### 5.4.1. Data Set Size

One of the simplest changes to make is in the size of the training data set. For rapid prototyping, a small set of ∼20 samples per unique source combination was used to optimise the workflow. This reduces to an average of just 10 samples after splitting spectra into training and validation sets. With the extremes in gain shifts, total counts, and relative activity of the mixed sources, this makes for a data set that cannot possibly represent the diverse range of spectra that may be present in experimental test sets. As a brief experiment, the same parameters used to generate the unshielded data set of the reference model were applied here.

**Figure 8:**
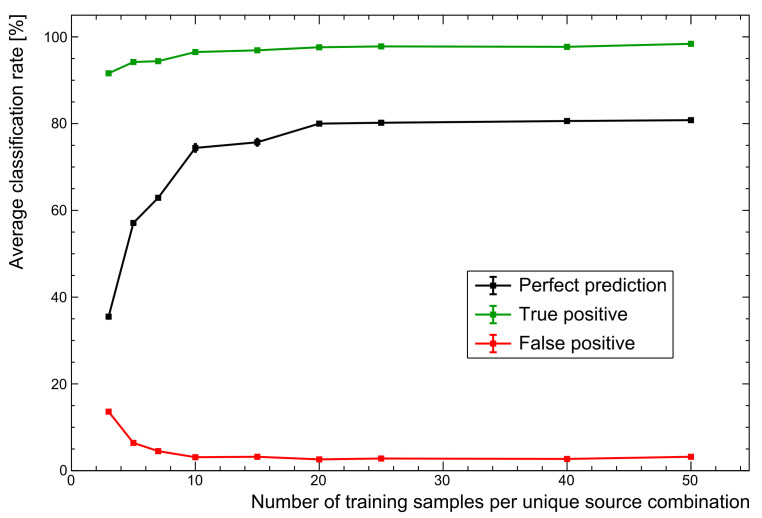
Comparison of performance with number of training samples. While there is a sharp rise in performance as more training spectra are added, the performance plateaus for these particular data. For more complex data sets, additional training samples are expected to become far more important.

As Figure 8 shows, changing nothing but the size of the training set sees PPR performance increase to ∼81%. The advantages of a larger set are seen to plateau here because the training data variations are all well represented. Of course, the limitation here is that training data are entirely simulated, missing some of the the subtleties of real spectra. However, this general trend does demonstrate why augmenting particularly small training sets with simulated data is very often a worthwhile endeavour. The next section explores the inclusion of real spectra as a more realistic use of the data likely to be available when deploying systems.

#### 5.4.2. Including Real Data

Simulated data are only ever an approximation of reality. The generalised approach using only simulated training data provided a rapid development environment, but they can exclude the subtleties of real spectra. Ideally, all training data would be experimentally obtained, but a compromise often made is to supplement the available on-site data with those of a simulation.

New training sets were generated to contain an even mix of real and simulated point source data at 10 cm, averaging 10 samples per source combination for direct comparison with the reference model. The PPR averaged an excellent 89.8(9)%, with ROC curves showing that the ${}^{44}$Ti, ${}^{22}$Na, and ${}^{60}$Co sources were, once again, the cause of more incorrect predictions. Including as much real data as possible is, therefore, highly recommended, whatever the application, and these results clearly demonstrate the advantages of doing so for the purposes of RIID.

#### 5.4.3. Generalisation

These models have already been shown to perform relatively well at different stand-off distances and levels of shielding. In each case, the model was trained on a specific simulated data set, and tested on the experimental version. However, many data sets may be combined to make the single, generalised model that real world applications need. A large data set was made by combining every individual data set, each with 10 training samples per source combination. For the sake of comparison, the training data were entirely simulated. The reference model was then trained on this all-inclusive set. This generalised model was finally used to make predictions on all of the test sets separately for direct comparison with the performance of the previous sections (Table 5).

The results across all the sets are much improved, even exceeding the addition of real spectra for the 10 cm stand-off data set. This follows expectations, given that the training data here are 6× larger and far more comprehensive. Challenging conditions still make heavy shielding and close geometries more difficult, mirroring the trends seen with the more focused models trained on individual data sets. However, there is clearly scope for training a generalised model to handle spectra produced under all kinds of external challenges, on top of complications present in every spectrum as a result of the physical detection mechanisms.

## 6. Conclusions

This work demonstrates that even basic CNNs may be used for the rapid, accurate identification of multi-isotope sources. The focus of this work was the methodology and performance of a simple model when applied to challenging data sets, even where experimental data are not readily available. A sequential CNN was applied to the RIID problem space to identify arbitrary mixtures of up to nine nuclides. Precision and the AUC metric are often too close for meaningful comparisons, so taking the full set of possible sources, ‘perfect’ classification rates were used as a complementary performance metric. An average perfect rate of ∼74% was seen for uncalibrated experimental spectra that contained large gain shifts, low energy thresholds, often poor statistics, and were trained exclusively on a small sample of simulated data.

Applying a consistent model architecture to the conditions that most challenge RIID showed comparable performance in most cases. In the extremes, it was seen that predictions faltered for heavily shielded isotopes, primarily those with low energy emissions, due to a loss of critical features in the spectra. In close geometries, multi-isotope spectra become far more complex due to extra summation between photon emissions. Despite this, models tested on the 1 cm stand-off data still performed well.

The inclusion of many additional training spectra raised perfect prediction rates to ∼81% in a trend typical of deep learning problems. The variation in training set characteristics demonstrates a strong influence on performance, so evaluation of models for RIID should not be confined to just the CNN architecture and its hyperparameters. Indeed, most real-world applications require representation of many different scenarios to account for geometries, crystal sizes, intrinsic resolutions, and any other physical effect that will distort the profile of a spectrum. The importance of representation is highlighted when real spectra are mixed into the pool of training samples. The perfect prediction rate for these same test sets used for the reference model was increased by ∼15%, a trend that the work of Ref. [19] also noted for a similar application. Lastly, this work also indicates that CNN-based models lend themselves particularly well to a generalised RIID model that can accommodate a range of conditions expected in the deployment of real systems.

To summarise, this work shows that relatively simple CNN models can be successfully applied to pragmatic nuclide identification. A constantly evolving landscape of deep learning techniques often produces new and advanced convolution architectures. These, combined with the domain knowledge necessary for effective pre-processing in gamma spectrometry, may prove ideal for rapid and robust real-time CNN-based RIID systems.

## 7. Future Work

Current efforts seek to combine further data sets for the purpose of training a general purpose model. The preliminary results presented demonstrate the potential for such a model. Fortunately, the techniques outlined in this work are extremely flexible, allowing the expansion of training sets to combinations of any nuclides and conditions that may be simulated. The next step for this work is to take the methodology that was developed here and apply it to new and more varied experimental data. However, it must be noted that scalability of a single model in this way is limited. Future work, therefore, should also seek to compare performance to a modular ensemble network. Instead of a single model, there would be a combination of nine separate CNNs performing a binary classification for each source, the results of which are brought together later, similar to the approach of Ref. [10].

## Figures and Tables

**Table 1:** Simple binary classification outcomes.

		Actual Label	
		1	0
Prediction	1	True Positive (TP)	False Positive (FP)
	0	False Negative (FN)	True Negative (TN)

**Table 2:** Relevant reference model hyperparameters.

Parameter	Value
Optimiser	Adam
Initialisers (kernel, bias)	He normal, zeros
Activation function	Leaky ReLU (α=0.1)
Batch size	32
Dense layer nodes	700
Dropout (spatial, dense)	0.5, 0.5
Filters per Conv. layer	40
Filter size	7
Pooling type	Max pooling (length 2, stride 2)
Padding	Same
Loss function	Binary cross-entropy

**Table 3:** Overall classification rates for the reference model.

		Actual	
		1	0
Predicted	1	96.5(1)%	3.1(2)%
	0	3.5(1)%	96.9(2)%

**Table 4:** Average perfect classification rates across data sets. All models were trained using the reference model architecture. Reference model developed on marked data set (*).

Stand-Off Data	AUC	Perfect Rate [%]	Shielded Data	AUC	Perfect Rate [%]
Point, 10 cm *	0.996 (1)	74.4 (9) *	2 cm Al	0.994 (1)	73.9 (9)
Point, 5 cm	0.995 (1)	73.9 (6)	4 cm Al	0.986 (2)	59.9 (7)
Point, 1 cm	0.991 (2)	66.5 (5)	6 cm Al	0.951 (4)	34.9 (8)

**Table 5:** Average perfect classification rates for all scenarios. The generalised model is compared with those trained individually on each set. Reference model developed on marked data set (*).

Test Set	Individual (%)	Generalised (%)
1 cm stand-off	66.5 (5)	72.6 (9)
5 cm stand-off	73.9 (6)	90.5 (4)
10 cm stand-off *	74.4 (9) *	90.9 (4)
1 Al plate	73.9 (9)	88.5 (7)
2 Al plates	59.9 (7)	81.5 (6)
3 Al plates	34.9 (8)	60.4 (7)

## Data Availability

Not applicable.

## Abbreviations

The following abbreviations are used in this manuscript:

RIID	Radio-Isotope IDentification
CNN	Convolutional Neural Network
ANN	Artificial Neural Network
ROC	Receiver Operator Characteristic
TPR	True Positive Rate
FPR	False Positive Rate
PPR	Perfect Performance Rate
AUC	Area Under Curve
